# Asymmetric Synthesis and Absolute Configuration Assignment of a New Type of Bedaquiline Analogue

**DOI:** 10.3390/molecules201219846

**Published:** 2015-12-11

**Authors:** Chang-Jiang Qiao, Xiao-Kui Wang, Fei Xie, Wu Zhong, Song Li

**Affiliations:** 1 School of Pharmaceutical Engineering, Shenyang Pharmaceutical University, 103 Wenhua Road, Shenyang 110016, China; qchj2004@163.com; 2 Laboratory of Computer-Aided Drug Design & Discovery, Beijing Institute of Pharmacology and Toxicology, 27 Taiping Road, Beijing 100850, China; wxkcaptain@163.com (X.W.); xiefei0058@aliyun.com (F.X.)

**Keywords:** antituberculosis, asymmetric synthesis, bedaquiline analogues, absolute configuration assignment

## Abstract

Bedaquiline is the first FDA-approved new chemical entity to fight multidrug-resistant tuberculosis in the last forty years. Our group replaced the quinoline ring with a naphthalene ring, leading to a new type of triarylbutanol skeleton. An asymmetric synthetic route was established for our bedaquiline analogues, and the goal of assigning their absolute configurations was achieved by comparison of experimental and calculated electronic circular dichroism spectra, and was confirmed by the combined use of circular dichroism and NMR spectroscopy.

## 1. Introduction

Tuberculosis is a widespread infectious disease that threatens one-third of the World population [1]. Most of these infections have no symptoms, a situation known as latent tuberculosis, and almost 10% of these latent infections will progress to the active disease [2]. The emergence of multidrug-resistant tuberculosis (MDR-TB) and extensive-drug resistant tuberculosis (XDR-TB) has invalidated the current commercially available medicines, such as isoniazid, rifampin, pyrazinamide, ethambutol and streptomycin [3]. Therefore, to stop the prevalence of tuberculosis, new drugs with novel mechanisms of action are urgently needed.

Bedaquiline, which targets the adenosine triphosphate synthase (ATPase) *M. tuberculosis* [4], was approved by the FDA to specifically treat MDR-TB. Because of its novel mechanism, bedaquiline does not have cross-resistance with current antituberculosis drugs. Many efforts have been made by scientists to locate the exact binding domain of bedaquiline in *M. tuberculosis* ATPase [5,6,7,8,9]. Most recently, in 2015, the X-ray crystal structures of a mycobacterial C_9_ ring with and without a bedaquiline molecule were reported, and it was suggested that bedaquiline blocked the ion-binding sites of the *c*-subunits [10].

During the development of bedaquiline, the structure-activity relationship (SAR) has always been the focus of scientists’ attention. Originally, the group led by Koen Andries at Janssen Pharmaceutica selected bedaquiline from more than 200 analogues [11] and, later, from over 500 compounds with a modulation on the aliphatic side chain; a total of 200 compounds with increased flexibility around the two stereogenic centers were synthesized and tested against Gram-positive bacteria to expand the diarylquinoline’s (DARQ’s) antibacterial spectrum [12]. Another group also greatly contributed to probing the SAR of bedaquiline by dividing bedaquiline into four quarters and modifying every quarter to evaluate the biologically active motifs [13,14,15,16,17].

**Figure 1:**
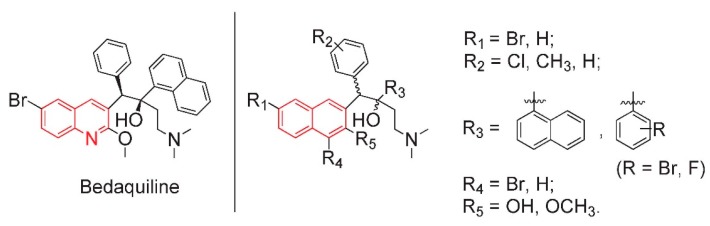
Structure of bedaquiline and our designed analogues of the TARB skeleton.

All the above modifications were based on the DARQ skeleton, but there is no confirmed proof that the quinoline ring is indispensable for anti-tuberculosis activity. Therefore, our group attempted to replace the quinoline ring with a naphthalene ring. As a result, the DARQ skeleton was changed into a triarylbutanol (TARB) skeleton. A series of substituted TARB compounds were synthesized and evaluated for MIC_90_, and most of them exhibited a comparable or even better inhibitory effect against *M. tuberculosis H37Rv* than bedaquiline (Figure 1) [18]. In accordance with Koen Andries’s results, the (1*R*, 2*S*) and (1*S*, 2*R*) mixture was superior to the (1*S*, 2*S*) and (1*R*, 2*R*) one.

To continue our research on determining which type of optical isomer of our analogues played a critical role in antituberculosis activity, we need to build a general asymmetric synthetic route to generate the four respective optical isomers and assign their absolute configurations (ACs).

## 2. Results and Discussion

### 2.1. Synthesis of Four Optical Isomers

**Scheme 1:**
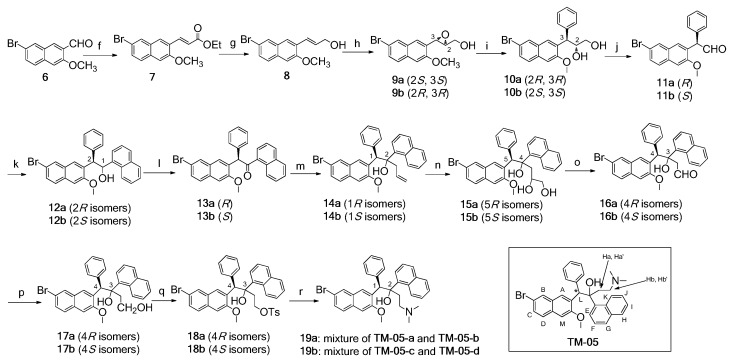
Asymmetric synthesis of four isomers of **TM-05**. *Reagents and conditions*: (**f**) NaHMDS, (EtO)_2_P(O)CH_2_CO_2_Et, THF, 0 °C to r.t., 2 h, 87%; (**g**) DIBAL-*H*, CH_2_Cl_2_, r.t., 2 h, 76%; (**h**) Ti(OiPr)_4_, (+)-DIPT, ^t^BuOOH, 4 Å zeolite, CH_2_Cl_2_, −20 °C, 4 h, 60%; (**i**) PhMgBr, CuI, THF, −20 °C, 3 h, 60%; (**j**) NaIO4, CH_2_Cl_2_, r.t., 3 h; (**k**) 1-naphthylmagnesium bromide, THF, 0 °C, 2 h, 62% over 2 steps; (**l**) DMP, CH_2_Cl_2_, r.t., 2 h, 95%; (**m**) allylzinc bromide, THF, r.t., 5 min, 60%; (**n**) OsO4, NMO, THF/water/acetone (1:1:1), r.t., 8 h; (**o**) NaIO_4_, CH_2_Cl_2_, r.t., 3 h; (**p**) NaBH_4_, CH_3_OH, r.t., 3 h, 50% over 3 steps; (**q**) TsCl, DMAP, pyridine, r.t., 16h, 58%; (**r**) HNMe_2_, THF.

After a thorough investigation into the asymmetric synthesis of bedaquiline, we found that only two asymmetric synthetic routes were available, reflecting the difficulty of building two vicinal chiral centers at sterically hindered positions surrounded by three bulky aryl groups [19]. Because we needed to obtain all four types of optical isomers, we proposed our asymmetric synthetic route based on the research of Chandrasekhar’s group [20], starting with the synthesis of **TM-05** as a starting material (Scheme 1).

**Scheme 2:**
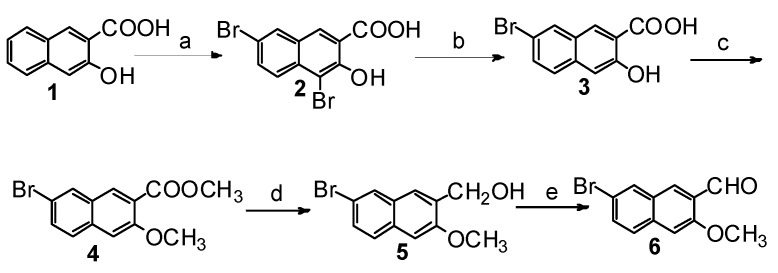
Synthesis of naphthaldehyde **6**. *Reagents and conditions*: (**a**) Br_2_, HOAc, reflux, 3 h, 91%; (**b**) tin powder, concentrated HCl, HOAc, reflux, 3 h, 93%; (**c**) K_2_CO_3_, (CH_3_O)_2_SO_2_, acetone, reflux, 10 h, 86%; (**d**) LiAlH_4_, THF, r.t., 8 h, 78%; (**e**) DMP, CH_2_Cl_2_, r.t., 1 h, 95%.

To prepare the desired starting material naphthaldehyde **6** (Scheme 2), 3-hydroxy-2-naphthoic acid (**1**) was first brominated by excess bromine at positions 4 and 7, followed by selective debromination with tin powder in an acidic environment to generate the 7-monobromo-substituted naphthoic acid **3** [21]. Treatment of naphthoic acid **3** with the methylation reagent dimethyl sulfate yielded the methoxy ester compound **4**, which was reduced by LiAlH_4_ to give the corresponding methoxy alcohol compound **5**. After oxidation of **5** using Dess-Martin periodinane (DMP) reagent, naphthaldehyde **6** was obtained in a high overall yield of 54% over five steps.

After we obtained starting material **6**, we followed Chandrasekhar’s procedure special attention to the problem of the low solubility of compounds **13a** and **13b**. *Trans*-allyl alcohol **8** as a Sharpless epoxidation standard substrate was easily regioselectively synthesized from **6** by Horner-Wadsworth-Emmons olefination and subsequent reduction with DIBAL-*H*. Asymmetric epoxidation is a critical step in controlling the final products’ optical purity. Active powdered 4 Å molecular sieves made *trans*-allyl alcohol **8** convert completely into epoxy alcohol **9a** with 87% *e.e.* when using l-(+)-diisopropyl tartrate (DIPT) and to **9b** with 97% *e.e.* when using d-(−)-DIPT [22].

Once the epoxy ring of epoxy alcohol **9a** was regioselectively opened in the presence of active CuI and freshly prepared Grignard reagent [23], the stereochemistry of the biaryl-substituted tertiary carbon of diol **10a** would remain unchanged. Cleavage of the diol was effectively and conveniently performed by treatment with NaIO_4_-impregnated silica gel to convert the diol **10a** into the aldehyde **11a** [24]. The introduction of the third aryl group to form the TARB skeleton was achieved through the attack of 1-naphthylmagnesium bromide to aldehyde **11a**, followed by oxidation with DMP to yield the keto compound **13a**.

After the first chiral center was enantioselectively constructed in **9a**, the diastereomers of **14a** resulting from the second chiral center could be differentiated by physical properties, such as their molecular polarity which facilitates the isolation process. Therefore, the keto compound **13a** was converted to the allylic compound **14a** without any stereoselective control. Compound **13a** has a very low solubility in most commonly used solvents, such as dichloromethane, THF and ethyl acetate. This physical property is very different from the synthetic intermediate of bedaquiline, and created a large obstacle for the next step because we needed to use as little as possible of the solvent THF for allylation [25]. Finally, allylation with freshly prepared allylzinc bromide [26] was achieved using crude product **13a** without purification. The classic method for the oxidative cleavage of olefin compounds using OsO_4_ and NaIO_4_ was applied to **14a** [27], affording the triaryl aldehyde **16a**. After the triaryl aldehyde **16a** was reduced to an alcohol by NaBH_4_, *O*-tosylation and displacement of the tosyl group with dimethylamine were conducted to produce the mixture **19a**.

To obtain optical purity-enriched final isomers, we made many attempts to investigate the proper isolation steps of **14a**, **16a**, **17a**, **18a** and **19a**, excluding compound **15a** due to the complexity of dealing with three chiral centers. Eventually, we found that compound **19a** gave the best result when using dichloromethane as the eluent for glass column silica gel chromatography. For the sake of convenience, we defined the upper spot on the TLC plate (CH_2_Cl_2_/MeOH = 50:1) as **TM-05-a** (Rf = 0.3), and the lower spot as **TM-05-b** (Rf = 0.25). The epoxy alcohol **9b** went through the same synthetic procedures as **9a**, and compound **19b** was thus obtained. Regarding **19b**, the upper spot **TM-05-c** (Rf = 0.3, CH_2_Cl_2_/MeOH = 50:1) and the lower spot **TM-05-d** (Rf = 0.25, CH_2_Cl_2_/MeOH = 50:1) were defined.

### 2.2. Determination of Optical Purity

According to the Sharpless epoxidation empirical rule [28], compound **9a** should be (2*S*, 3*S*) enriched in the enantiomer, whereas compound **9b** should be enriched in the (2*R*, 3*R*) one. However, their absolute configurations needed to be verified, as discussed in Section 2.3. Their optical purities were determined by chiral HPLC, and the HPLC spectra are available in the Supplementary Materials. Compound **9a** had an *e.e.* value of 87%, while compound **9b** had a much higher *e.e.* value of 97%. Because, the stereochemistry of the biaryl-substituted tertiary carbons in **19a** and **19b** stemmed from the epoxy alcohols **9a** and **9b**, the four isomers after isolation should have the same optical purity as that of **9a** and **9b**, respectively. This speculation was firmly confirmed by the optical purity of **TM-05-a** (*e.e.* = 87%) and its enantiomer **TM-05-c** (*e.e.* = 97%). Therefore, based on these highly coherent data, we claim that **TM-05-b** possessed an *e.e.* value of 87% and **TM-05-d** 97%.

### 2.3. Absolute Configuration Assignment 

#### 2.3.1. AC Assignment by Electronic Circular Dichroism (ECD) Spectroscopy

Because every final optical isomer of **TM-05-a**–**TM-05-d** has a heavy atom, bromine, anchored to the naphthalene ring, their ACs could be determined if two single crystals from two group of enantiomers were obtained. However, all of our attempts at growing single crystals failed. While we were searching for other methods to assign ACs, ECD caught our attention. In recent years, ECD spectroscopy has been proven reliable and has been broadly used for the determination of the ACs of chiral compounds, by simply matching the calculated ECD curve with the experimental one [29]. Three aromatic chromophores surrounding the two chiral carbons make it possible to apply the ECD method to our AC analysis.

**Figure 2:**
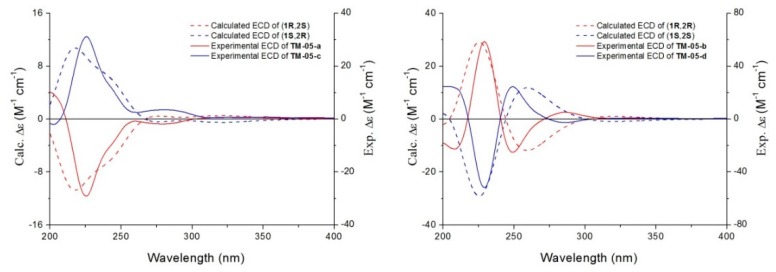
Comparison of experimental ECD spectra and calculated ECD spectra of the four **TM-05** optical isomers.

As shown in Figure 2, we observe a good match between **TM-05-a** and (1*R*, 2*S*), **TM-05-b** and (1*R*, 2*R*), **TM-05-c** and (1*S*, 2*R*) and **TM-05-d** and (1*S*, 2*S*). The experimental ECD spectra were recorded at a concentration of 0.2−1.0 mg/mL in methanol, and the quantum chemical calculations were only conducted for two diastereomers, (1*R*, 2*S*) and (1*R*, 2*R*), because the calculated ECD spectra of their respective enantiomers could be easily plotted by showing mirror symmetry along the wavelength axis. Computational details are described in the following experimental section. According to these satisfactorily matched ECD spectra, (1*R*, 2*S*), (1*R*, 2*R*), (1*S*, 2*R*) and (1*S*, 2*S*) were assigned to **TM-05-a**–**TM-05-d**, respectively.

#### 2.3.2. AC Corroboration by the Combined Use of Circular Dichroism and NMR Spectroscopy

In spite of the agreement achieved between the computational and experimental ECD spectra, the ECD method is not 100% accurate because the calculated ECD spectra is only a simulation and cannot reflect all of the actual molecular properties in the solvent [30]. Therefore, the above results need to be corroborated.

**Figure 3:**
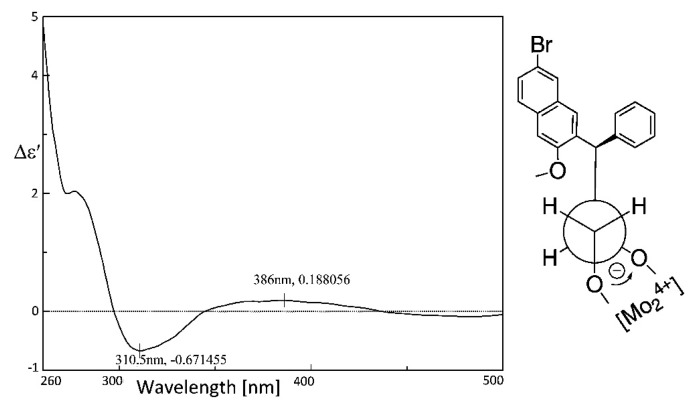
Induced CD spectra of *in situ* formed Mo-complex of **10a**.

To confirm the calculated results, we tried to assign the ACs step-by-step. First, we tested the Mo_2_(OAc)_4_-induced CD spectrum of compound **10a** according to the previously described procedure [31], specifically used for the AC assignment of optically active 1,2-diols. According to the empirical rule proposed by Snatzke [32], the negative Cotton effect IV at *ca.* 310 nm observed in the Mo_2_(OAc)_4_-induced CD (DMSO) spectrum reflects a negative torsional angle (OH)-C-C-(OH). For steric reasons, the conformation of the diol **10a** prefers the bulky diaryl group pointing away from the remaining acylate moieties. As shown in Figure 3, the 2*R* configuration in **10a** was concluded, and, considering the phenyl located on the reverse side of the secondary hydroxyl group, the AC of **10a** can be defined as (2*R*, 3*R*), as expected when using l-(+)-(DIPT). Actually, we also tried to grow single crystals for **10a** and **10b**, but unfortunately both turned out to be the racemate of two stereoisomers in acetone. CCDC-1417941 and CCDC-1417940 contains the supplementary crystallographic data for **10a** and **10b**. These data can be obtained free of charge via http://www.ccdc.cam.ac.uk/conts/retrieving.html (or from the CCDC, 12 Union Road, Cambridge CB2 1EZ, UK; Fax: +44 1223 336033; E-mail: deposit@ccdc.cam.ac.uk). Crystal structures of the two corresponding stereoisomers were attached in Supplementary Materials (Figure S10).

**Figure 4:**
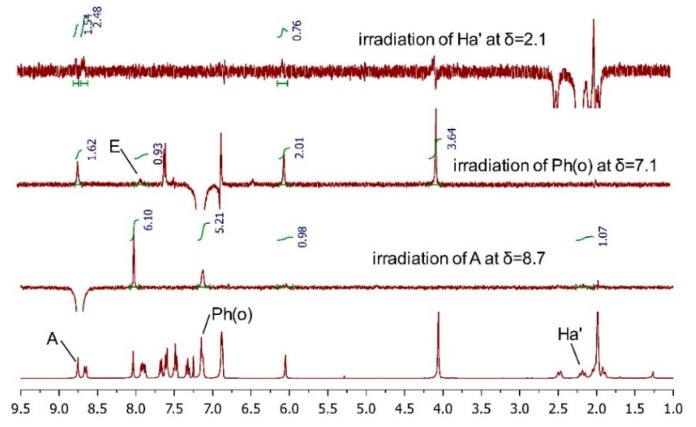
1D NOESY spectra of **TM-05-c**.

**Figure 5:**
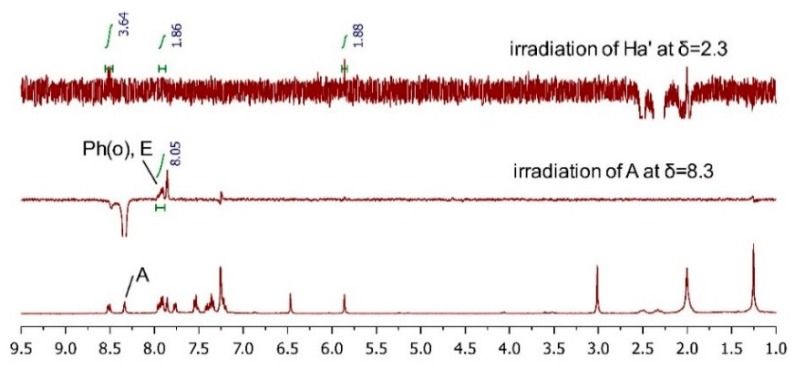
1D NOESY spectra of **TM-05-d**.

Based on the Sharpless epoxidation empirical rule and the Mo_2_(OAc)_4_-induced CD spectrum result, compound **10a** was unequivocally assigned as (2*R*, 3*R*) and **10b** was supposed to be (2*S*, 3*S*). To further clarify the AC of the final isomers, ^1^H-NMR and 2D NMR (NOESY or ROESY) experiments were performed on **TM-05-c** and **TM-05-d** and the proton chemical shift values and coupling constants of **TM-05-c** and **TM-05-d** are summarized in Table 1. Because the structural difference between bedaquiline and our final compounds is so small and according to the overlay results of the crystal structure of bedaquiline from the PDB database (ID: 4V1F) and the optimized conformer of (1*R*, 2*S*) isomer of **TM-05** (Figure S9), it is reasonable to assume that they will have similar characteristic correlation peaks in their 2D NOESY or ROESY spectra. Specifically, in the spectra of the (1*R*, 2*S*)/(1*S*, 2*R*) group of **TM-05**, the Ph(o)/E and A/Ha′ correlations should be observed, and the Ph(o)/Ha′ and A/E correlations should be absent; conversely, regarding the (1*R*, 2*R*)/(1*S*, 2*S*) group of **TM-05**, the Ph(o)/Ha′ and A/E correlations should appear, while Ph(o)/E and A/Ha′ should disappear [33]. However, in the 2D NOESY spectrum (Figure S5) of **TM-05-c** and the 2D ROESY spectrum (Figure S6) of **TM-05-d**, the respective characteristic correlation peaks were submerged in the noise signals. Therefore, 1D NOESY experiments at specific proton chemical shifts were carried out. For **TM-05-c** (Figure 4, full spectra are in the Supplementary Materials), irradiation of the Ph(o) and H_a_′ protons induced an enhancement of the integration of proton E by 0.93% and proton A by 1.54%, respectively, and it is noteworthy that the irradiation of proton A did not affect proton E. For **TM-05-d** (Figure 5), irradiation of the H_a_′ proton increased the integration of proton Ph(o) by 1.86%, and irradiation of proton A greatly enhanced proton E and Ph(o) by 8.05%, indicating that protons A, E and Ph(o) are sterically close. These 1D NOESY results matched perfectly with the characteristic correlations mentioned above; therefore, we concluded that **TM-05-c** belonged to the (1*R*, 2*S*)/(1*S*, 2*R*) group of **TM-05**, and **TM-05-d** belonged to the (1*R*, 2*R*)/(1*S*, 2*S*) group. Considering the AC of the tertiary carbon defined by the Mo_2_(OAc)_4_-induced CD spectra, the ACs of **TM-05-a**, **TM-05-b**, **TM-05-c** and **TM-05-d** were unambiguously determined as (1*R*, 2*S*), (1*R*, 2*R*), (1*S*, 2*R*) and (1*S*, 2*S*), respectively, which are in accordance with the ECD method results.

**Table 1:** Proton chemical shifts and coupling constants in CDCl_3_ of **TM-05-c** and **TM-05-d.**

Compound TM-05-c			Compound TM-05-d		
Proton	δ (ppm)	*J* (Hz)	Proton	δ (ppm)	*J* (Hz)
N(CH_3_)_2_,H_b_,H_b_’	1.88–2.04	m	N(CH_3_)_2_,H_b_,H_b_’	1.93–2.06	m
H_a_′	2.17	t (12.9)	H_a_’	2.32	t (12.0)
H_a_	2.47	d (14.3)	H_a_	2.49	d (14.3)
OCH_3_	4.05	s	OCH_3_	3.00	s
L	6.04	s	L	5.86	s
Ph(m, p)	6.86–6.88	m	M	6.46	s
Ph(o),M	7.12–7.14	m	Ph(p),F,G,D	7.20–7.25	m
F	7.31	t (7.6)	Ph(m),H	7.34–7.42	m
I,C	7.45–7.50	m	C,J	7.51–7.55	m
D,J	7.57–7.61	m	I	7.77	d (8.1)
G	7.67	d (8.1)	B	7.86	s
E,H	7.87–7.92	m	Ph(o),E	7.91–7.97	m
B	8.03	s	A	8.34	s
K	8.65	d (8.7)	K	8.52	d (8.7)
A	8.75	s			

## 3. Experimental Section

### 3.1. General Information

Unless otherwise noted, all reactions were performed under an atmosphere of nitrogen. Except for l-(+)-DIPT, d-(−)-DIPT and Ti(OiPr)_4_ from Sigma-Aldrich Co. Ltd (Beijing, China), all other reagents and solvents were purchased from common commercial suppliers. Only THF and CH_2_Cl_2_ needed to be distilled according to standard procedures: CH_2_Cl_2_ by CaH_2_ and THF by LiAlH_4_. NMR spectra were recorded on a Bruker ARX400 spectrometer (Bruker Inc., Karlsruhe, Germany) with Me_4_Si as an internal standard. Mass spectra were obtained on an 1260-G6230A LC-MS spectrometer (Agilent, Santa Clara, CA, USA). ECD spectra were recorded in a quartz cuvette with a 1-mm optical path length using a Jasco J-815 CD spectrometer (Jasco Inc., Tokyo, Japan). All quantum computations were performed using the Gaussian 09 program package on an IBM cluster machine located at the High Performance Computing Center of Peking Union Medical College.

### 3.2. Computational Details

We selected the (1*R*, 2*S*) and (1*R*, 2*R*) configurations for quantum chemical calculations. The initial conformation search was carried out using the MMFF94 molecular mechanics force field via the MOE software package. The resulting eight conformers (Figure S7) for (1*R*, 2*S*) and six conformers (Figure S8) for (1*R*, 2*R*), within a 5-kJ/mol window, were optimized in the framework of density functional theory (DFT) using the Becke 3-Lee-Yang-Parr (B3LYP) exchange-correlation functional at the 6-31G (d) basis set level (see supporting information). All of these conformers were verified as the true minima of the potential energy surface by showing no imaginary frequency. Solvent effects of methanol were considered by using the polarizable continuum model (PCM). Oscillator strengths and rotational strengths in both dipole length (R_len_) and dipole velocity (R_vel_) representations of the 60 lowest electronic transitions were calculated for each conformer. Every conformer’s ECD spectra was simulated by using a Gaussian function with R_vel_ and a bandwidth σ = 0.45 eV. Equilibrium populations of conformers at 298.15 K were calculated from their relative free energies (ΔG) using Boltzmann statistics. The final ECD spectra were obtained by averaging all the simulated ECD spectra of the calculated conformers according to Boltzmann distribution.

### 3.3. Synthesis and Characterization

*4,7-Dibromo-3-hydroxy-2-naphthoic acid* (**2**). At room temperature, a suspension of 3-hydroxy-2-naphthoic acid **1** (50 g, 266 mmol) in acetic acid (500 mL) was mechanically stirred for 10 min. A solution of bromine (34 mL, 665 mmol) in acetic acid (200 mL) was then added dropwise through a dropping funnel. Then the mixture was heated to reflux for 3 h. After cooling to room temperature, the reaction was quenched with 3000 mL of ice water. The yellow solid was filtered and dried to give a crude product of **2** (84 g, 91%) as a yellow solid. ^1^H-NMR (400 MHz, DMSO-*d*_6_): δ = 3.63 (br. s), 7.84 (dd, *J* = 9.2, 2.0 Hz, 1H), 7.98 (d, *J* = 9.0 Hz, 1H), 8.41 (d, *J* = 2.0 Hz, 1H), 8.63 (s, 1H) ppm. HRMS (ESI): [M − H]^−^, found 344.8591. C_11_H_5_Br_2_O_3_^−^ requires 344.8585.

*7-Bromo-3-hydroxy-2-naphthoic acid* (**3**). Compound **2** (70 g, 202 mmol), tin powder (23.9 g, 202 mmol) and 250 mL of concentrated HCl were successively added into acetic acid (800 mL). The mixture was heated to reflux for 3 h. After cooling to room temperature, the reaction was quenched with ice water (1000 mL) and the solid was filtered and dried to afford **3** (50 g, 93%) as a yellow solid. ^1^H-NMR (400 MHz, DMSO-*d*_6_): δ = 3.37 (br. s), 7.37 (s, 1H), 7.64 (dd, *J* = 9.0, 2.0 Hz, 1H), 7.76 (d, *J* = 9.0 Hz, 1H), 8.28 (d, *J* = 2.0 Hz, 1H), 8.54 (s, 1H) ppm. HRMS (ESI): [M − H]^−^, found 264.9502. C_11_H_6_BrO_3_^−^ requires 264.9500. 

*Methyl 7-bromo-3-methoxy-2-naphthoate* (**4**). To a solution of **3** (200 g, 750 mmol) in acetone (2000 mL), anhydrous K_2_CO_3_ (757 g, 5475 mmol) and dimethyl sulfate (236 g, 1872 mmol) were added at room temperature with mechanical stirring. After refluxing for 10 h, the reaction was cooled to room temperature. The insoluble K_2_CO_3_ was filtered and washed with EtOAc. The combined filtrate was concentrated under reduced pressure and extracted with EtOAc and water. The combined organic layer was washed with water and brine, dried with Na_2_SO_4_ and rotary evaporated to give **4** (190 g, 86%) as a pale yellow solid. ^1^H-NMR (400 MHz, CDCl_3_): δ = 3.95 (s, 3H), 3.99 (s, 3H), 7.17 (s, 1H), 7.57 (dd, *J* = 8.7, 2.0 Hz, 1H), 7.61 (d, *J* = 8.7 Hz, 1H), 7.96 (d, *J* = 2.0 Hz, 1H), 8.18 (s, 1H) ppm. HRMS (ESI): calcd. for C_13_H_11_BrO_3_Na^+^ [M + Na]^+^ 316.9789, found 316.9785.

*7-Bromo-3-methoxy-2-naphthalenemethanol* (**5**). To a suspension of LiAlH_4_ (8.36 g, 220 mmol) in THF (100 mL), a solution of **4** (59 g, 200 mmol) in THF (500 mL) was added dropwise via a dropping funnel at room temperature. After stirring for 8 h, water (20 mL) was added dropwise with vigorous stirring to quench the reaction. The mixture was filtrated through a plug of Celite and rinsed with EtOAc. The combined filtrate was dried with Na_2_SO_4_ and concentrated to give **5** (46 g, 78%) as a white solid. ^1^H-NMR (400 MHz, CDCl_3_): δ = 3.97 (s, 3H), 4.82 (s, 2H), 7.09 (s, 1H), 7.50 (dd, *J* = 8.8, 2.0 Hz, 1H), 7.60 (d, *J* = 8.8 Hz, 1H), 7.65 (d, *J* = 2.0 Hz, 1H), 7.9 (s, 1H) ppm. GC-MS *m/z*: 266 (M^+^).

*7-Bromo-3-methoxy-2-naphthaldehyde* (**6**). To a solution of **5** (10 g, 37.4 mmol) in CH_2_Cl_2_ (150 mL) at 0 °C, Dess-Martin periodinane (DMP: 24 g, 56.6 mmol) was added slowly. After stirring for 1 h at room temperature, the reaction mixture was quenched with saturated aqueous Na_2_S_2_O_3_ (50 mL) and saturated aqueous NaHCO_3_ (50 mL). The aqueous phase was extracted with CH_2_Cl_2_. The combined organic layer was washed with water and brine, dried with Na_2_SO_4_ and rotary evaporated. The crude product underwent silica gel chromatography (eluent: EtOAc/hexane = 1:10) to yield **6** (9.4 g, 95%) as a yellow solid. ^1^H-NMR (400 MHz, DMSO-*d*_6_): δ = 4.01 (s, 3H), 7.58 (s, 1H), 7.72 (dd, *J* = 8.8, 2.0 Hz, 1H), 7.86 (d, *J* = 8.8 Hz, 1H), 8.35 (m, 2H), 10.46 (s, 1H) ppm. GC-MS *m/z*: 264 (M^+^).

*Ethyl (E)-3-(7-bromo-3-methoxynaphthalen-2-yl)acrylate* (**7**). To a solution of triethyl phosphonoacetate (6.78 g, 30.3 mmol) in THF (10 mL) at 0 °C, NaHMDS (14.2 mL, 28.4 mmol, 2.0 M in THF) was added slowly and stirred for 30 min at room temperature. A solution of **6** (5 g, 19 mmol) in THF (10 mL) was added dropwise to the above reaction mixture and then stirred for another 2 h. The reaction mixture was quenched with water, concentrated under reduced pressure and extracted with EtOAc. The combined organic layer was washed with water and brine, dried with Na_2_SO_4_, rotary evaporated and crystallized to give **7** (5.5 g, 87%) as a white solid. ^1^H-NMR (400 MHz, DMSO-*d*_6_): δ = 1.28 (t, *J* = 7.2 Hz, 3H), 3.98 (s, 3H), 4.22 (q, *J* = 7.0 Hz, 2H), 6.78 (d, *J* = 16.2 Hz, 1H), 7.47 (s, 1H), 7.62 (dd, *J* = 8.7, 2.0 Hz, 1H), 7.81 (d, *J* = 8.7 Hz, 1H), 7.94 (d, *J* = 16.2 Hz, 1H), 8.10 (d, *J* = 2.0 Hz, 2H), 8.33 (s, 1H) ppm. HRMS (ESI): [M + Na]^+^, found 357.0096. C_16_H_15_BrO_3_Na^+^ requires 357.0102.

*(E)-3-(7-Bromo-3-methoxynaphthalen-2-yl)prop-2-en-1-ol* (**8**). DIBAL-*H* (360 mL, 360 mmol, 1.0 M in toluene) was added to a solution of **7** (60 g, 180 mmol) in CH_2_Cl_2_ (400 mL) at 0 °C. After being stirred for 2 h at room temperature, the reaction mixture was quenched with saturated aqueous Rochelle’s salt (300 mL) at 0 °C and the resulting suspension was stirred vigorously for 4 h. The reaction mixture was filtrated and the aqueous phase was extracted with EtOAc. The combined organic layer was washed with water and brine, dried with Na_2_SO_4_ and rotary evaporated. The crude product underwent silica gel chromatography (eluent: EtOAc/hexane = 1:5) to afford **8** (40 g, 76%) as a pale yellow solid. ^1^H-NMR (400 MHz, DMSO-*d*_6_): δ = 3.92 (s, 3H), 4.18 (m, 2H), 4.96 (t, *J* = 5.6 Hz, 1H), 6.53 (dt, *J* = 5.0, 16.0 Hz, 1H), 6.91(d, *J* = 16.0 Hz, 1H), 7.36 (s, 1H), 7.51 (dd, *J* = 2.0, 8.7 Hz, 1H), 7.75 (d, *J* = 8.7 Hz, 1H), 7.99(s, 1H), 8.08(d, *J* = 2.0 Hz, 1H) ppm. 

*(2S,3S)-3-(7-Bromo-3-methoxynaphthalen-2-yl)oxiran-2-yl]methanol* (**9a**). *(2R,3R)-3-(7-Bromo-3-methoxy-naphthalen-2-yl)oxiran-2-yl]methanol* (**9b**) Ti(OiPr)_4_ (2.43 g, 8.5 mmol) was added to a suspension of powdered molecular sieves (4 Å, 2 g, 40% *w*/*w* based on substrate) in CH_2_Cl_2_ (30 mL). After stirring for 5 min, l-(+)-DIPT (2.4 g, 10.2 mmol) in CH_2_Cl_2_ (10 mL) was added, and the reaction mixture was then stirred for 30 min, followed by slow addition of a solution of **8** (5 g, 17 mmol) in 120 mL of CH_2_Cl_2_. The resulting suspension was stirred for another 40 min and anhydrous *tert*-butyl hydroperoxide (5.5 M in nonane, 6.2 mL, 34.1 mmol) was added at the same temperature and stirred for 4 h. After warming to 0 °C, 20% aqueous NaOH saturated with NaCl (30 mL) was added and the mixture was stirred for 1 h. The resulting slurry was filtered through a plug of Celite and rinsed thoroughly with CH_2_Cl_2_. The organic layer was separated and the aqueous layer was extracted with EtOAc. The combined organic layer was washed with water and brine, dried with Na_2_SO_4_ and rotary evaporated. The crude product underwent silica gel chromatography (eluent: EtOAc/hexane = 1:5) to produce **9a** (87% *e.e.*) as a white solid (3.1 g, 60%). ^1^H-NMR (400 MHz, DMSO-*d*_6_): δ = 3.10 (m, 1H), 3.58 (m, 1H), 3.80 (ddd, *J* = 2.8, 5.6, 12.6 Hz, 1H), 3.95 (s, 3H), 4.16 (d, *J* = 1.7 Hz, 1H), 5.05 (t, *J* = 5.9 Hz, 1H), 7.40 (s, 1H), 7.56 (dd, *J* = 2.0, 8.8 Hz, 1H), 7.63 (s, 1H), 7.79 (d, *J* = 8.8 Hz, 1H), 8.11 (d, *J* = 2.0 Hz, 1H) ppm. HRMS (ESI): [2 M + Na]^+^ , found 640.9968. C_28_H_26_Br_2_O_6_Na^+^ requires 640.9973.

**9b** (97% *e.e.*) was obtained with the same procedure as **9a** using d-(−)-DIPT. HPLC [Chiralpak IC (250 mm × 4.6 mm, 5 µ), IPA/hexane = 30%:70%, flow = 1.0 mL, T = 20 °C, λ = 225 nm, t_R_ = 7.1 min (**9b**), 9.2 min (**9a**)].

*(2R,3R)-3-(7-Bromo-3-methoxynaphthalen-2-yl)-3-phenylpropane-1,2-diol* (**10a**) *(2S,3S)-3-(7-Bromo-3-methoxynaphthalen-2-yl)-3-phenylpropane-1,2-diol* (**10b**). A suspension of freshly prepared CuI (6 g, 32 mmol) in THF (34 mL) was cooled to −20 °C, and PhMgBr (22 mL, 66 mmol, 3 M in Et_2_O) was added via syringe and stirred for 1 h. A solution of **9a** (2 g, 6.5 mmol) in THF (10 mL) was added and stirred. After 3 h, the reaction mixture was warmed to 0 °C and quenched by a saturated aqueous solution of NH_4_Cl (50 mL). After the reaction mixture was concentrated under reduced pressure and extracted with EtOAc, the combined organic layer was washed with water and brine, dried with Na_2_SO_4_ and rotary evaporated. The crude product underwent silica gel chromatography (eluent: EtOAc/hexane = 1:5) to obtain **10a** as a white solid (1.5 g, 60%). ^1^H-NMR (400 MHz, DMSO-*d*_6_): δ = 3.20 (m, 1H), 3.32 (m, 1H), 3.83 (s, 3H), 4.38 (m, 1H), 4.53 (d, *J* = 9.5 Hz, 1H), 7.10–7.33 (m, 6H), 7.50 (dd, *J* = 2.0, 8.7Hz, 1H), 7.71 (d, *J* = 9.0 Hz, 1H), 8.08 (d, *J* = 2.0 Hz, 1H), 8.12 (s, 1H) ppm. HRMS (ESI): [M + Na]^+^, found 409.0425. C_20_H_19_BrO_3_Na^+^ requires 409.0415.

**10b** was obtained with the same procedure as **10a** using **9b**.

*(2R)-2-(7-Bromo-3-methoxynaphthalen-2-yl)-1-(naphthalen-1-yl)-2-phenylethanol* (**12a**) *(2S)-2-(7-Bromo-3-methoxynaphthalen-2-yl)-1-(naphthalen-1-yl)-2-phenylethanol* (**12b**). To a solution of **10a** (4 g, 10.3 mmol) in CH_2_Cl_2_ (100 mL), NaIO_4_ impregnated over silica (33 g, 20 % *w*/*w* on silica) was added and stirred for 3 h at room temperature. The reaction mixture was filtrated and rotary evaporated to give the crude product **11a** which was used immediately for the next reaction. All of the above **11a** was dissolved in THF (22 mL), and freshly prepared naphthyl Grignard reagent (22 mL, 44 mmol, 2 M in THF) was added slowly at 0 °C and stirred for 2 h. A saturated aqueous solution of NH_4_Cl (50 mL) was added to quench the reaction, and the aqueous layer was extracted with EtOAc. The combined organic layer was washed with water and brine, dried with Na_2_SO_4_ and rotary evaporated. The crude product underwent silica gel chromatography (eluent: EtOAc/hexane = 1:10) to obtain **12a** as a white solid (3.1 g, 62% over 2 steps). 

**12b** was obtained with the same procedure as **12a** using **10b**.

*(R)-2-(7-Bromo-3-methoxynaphthalen-2-yl)-1-(naphthalen-1-yl)-2-phenylethanone* (**13a**) *(S)-2-(7-Bromo-3-methoxynaphthalen-2-yl)-1-(naphthalen-1-yl)-2-phenylethanone* (**13b**). To a solution of **12a** (6.65 g, 13.8 mmol) in CH_2_Cl_2_ (100 mL), Dess-Martin periodinane (DMP: 9 g, 21 mmol) was added slowly and stirred for 2 h. After the reaction finished, 30 mL of asaturated aqueous solution of Na_2_S_2_O_3_ and 30 mL of a saturated aqueous solution of NaHCO_3_ were added and stirred for 1 h. After the reaction mixture became clear, it was extracted by CH_2_Cl_2_ three times. The combined organic layer was washed with water and brine, dried with Na_2_SO_4_ and rotary evaporated. **13a** (6.3 g, 95%) was obtained without column chromatography. ^1^H-NMR (400 MHz, CDCl_3_): δ = 3.80 (s, 3H), 6.33 (s, 1H), 7.10 (s, 1H), 7.19 (s, 1H), 7.33–7.60 (m, 10H), 7.78 (d, *J* = 1.7 Hz, 1H), 7.84 (d, *J* = 8.1 Hz, 1H), 7.94 (d, *J* = 8.1 Hz, 1H), 8.05 (d, *J* = 7.3 Hz, 1H), 8.58 (d, *J* = 8.4 Hz, 1H). HRMS (ESI): [M + Na]^+^, found 505.0616. C_29_H_21_BrO_2_Na^+^ requires 505.0602.

**13b** was obtained with the same procedure as **13a** using **12b**.

*(1R)-1-(7-Bromo-3-methoxynaphthalen-2-yl)-2-(naphthalen-1-yl)-1-phenylpent-4-en-2-ol* (**14a**), *(1S)-1-(7-Bromo-3-methoxynaphthalen-2-yl)-2-(naphthalen-1-yl)-1-phenylpent-4-en-2-ol* (**14b**). After the freshly prepared allylzinc bromide (83 mmol) was concentrated under reduced pressure, a solution of **13a** (2 g, 4.15 mmol) in THF (as little as possible) was poured into the allylzinc bromide’s bottle and stirred for 5 min. A saturated aqueous solution of NH_4_Cl (50 mL) was added, and the aqueous layer was extracted with EtOAc. The combined organic layer was washed with water and brine, dried with Na_2_SO_4_ and rotary evaporated. The crude product underwent silica gel chromatography (eluent: EtOAc/hexane = 1:20) to obtain **14a** as a white solid (1.3 g, 60%).

**14b** was obtained with the same procedure as **14a** using **13b**.

*(4R)-4-(7-Bromo-3-methoxynaphthalen-2-yl)-3-hydroxy-3-(naphthalen-1-yl)-4-phenylbutanal* (**16a**), *(4S)-4-(7-Bromo-3-methoxynaphthalen-2-yl)-3-hydroxy-3-(naphthalen-1-yl)-4-phenylbutanal* (**16b**). To a solution of **14a** (3.5 g, 6.7 mmol) and *N*-methyl morpholine-*N*-oxide (NMO: 3.9 g, 33.5 mmol) in THF/water/acetone (1:1:1, 30 mL) at 0 °C, OsO_4_ (0.68 mL, 0.1 mmol, 4% in water) was added. The reaction was warmed to room temperature and stirred for 8 h. A saturated aqueous solution of Na_2_S_2_O_3_ (10 mL) was added and stirred for 30 min. The reaction mixture was concentrated under reduced pressure and extracted with EtOAc. The combined organic layer was washed with water and brine, dried with Na_2_SO_4_ and rotary evaporated to give crude product **15a** which was used immediately for the next reaction.

All of the above **15a** was dissolved in CH_2_Cl_2_, and NaIO_4_ impregnated over silica (25 g, 20% *w*/*w* on silica) was added and stirred for 3 h at room temperature. The reaction mixture was filtrated and rotary evaporated to give **16a** without column chromatography.

**16b** was obtained with the same procedure as **16a** using **14b**.

*(4R)-4-(7-Bromo-3-methoxynaphthalen-2-yl)-3-(naphthalen-1-yl)-4-phenylbutane-1,3-diol* (**17a**), *(4S)-4-(7-Bromo-3-methoxynaphthalen-2-yl)-3-(naphthalen-1-yl)-4-phenylbutane-1,3-diol* (**17b**). All of the above **16a** was dissolved in 30 mL of methanol, and NaBH_4_ (0.3 g, 8 mmol) was added slowly at 0 °C. After stirring at room temperature for 3 h, the reaction mixture was filtrated, rotary evaporated and passed through silica gel chromatography column (eluent: EtOAc/hexane = 1:10) to give **17a** (1.8 g, 50% over 3 steps). ESI-MS *m/z*: 526 [M]^+^.

**17b** was obtained with the same procedure as **17a** using **16b**.

*(4R)-4-(7-Bromo-3-methoxynaphthalen-2-yl)-3-hydroxy-3-(naphthalen-1-yl)-4-phenylbutyl 4-methyl-benzenesulfonate* (**18a**), *(4S)-4-(7-Bromo-3-methoxynaphthalen-2-yl)-3-hydroxy-3-(naphthalen-1-yl)-4-phenylbutyl 4-methylbenzenesulfonate* (**18b**). **17a** (1 g, 1.9 mmol) was dissolved in 38 mL of pyridine, and 4-dimethylaminopyridine (DMAP: 0.47 g, 3.8 mmol) and TsCl (3.2 g, 19 mmol) were added slowly at 0 °C. After stirring for 16 h at room temperature, the reaction mixture was quenched by 5 mL of water. Most of the pyridine was rotary evaporated and the rest of the reaction mixture was extracted with EtOAc. The combined organic layer was washed with water and brine, dried with Na_2_SO_4_ and rotary evaporated. The crude product underwent silica gel chromatography (eluent: EtOAc/hexane = 1:10) to obtain **18a** as a white solid (0.75 g, 58%). ESI-MS *m/z*: 698 [M + NH_4_]^+^.

**18b** was obtained with the same procedure as **18a** using **17b**.

*(1R,2S)-1-(7-Bromo-3-methoxynaphthalen-2-yl)-4-(dimethylamino)-2-(naphthalen-1-yl)-1- phenylbutan-2-ol* (**TM-05-a**) and *(1R,2R)-1-(7-bromo-3-methoxynaphthalen-2-yl)-4-(dimethylamino)-2-(naphthalen-1-yl)-1-phenylbutan-2-ol* (**TM-05-b**). A solution of **18a** (0.6 g, 0.9 mmol) in Me_2_NH (75 mL, 3 M in THF) was stirred at 45 °C for 24 h. The reaction mixture was rotary evaporated and passes through a silica gel chromatography column (eluent: EtOAc t/hexane = 1:5) to obtain the mixture of **TM-05-a** and **TM-05-b**, namely **19a** (0.4 g, 82%). Further purification of **19a** through a silica gel chromatography column (eluent: CH_2_Cl_2_) yielded the final products **TM-05-a** (0.15 g, 37.5%, Rf = 0.3, CH_2_Cl_2_/MeOH = 50:1, 87% *e.e.*) and **TM-05-b** (0.1 g, 25%, Rf = 0.25, CH_2_Cl_2_/MeOH = 50:1, 97% *e.e.*). The mixture of **TM-05-c** and **TM-05-d**, namely **19b** (0.42 g, 86%) was obtained with the same procedure as **19a**. 

*(1S,2R)-1-(7-Bromo-3-methoxynaphthalen-2-yl)-4-(dimethylamino)-2-(naphthalen-1-yl)-1- phenylbutan-2-ol* (**TM-05-c**). **TM-05-c** (0.12 g, 28.6%, Rf = 0.3, CH_2_Cl_2_/MeOH = 50:1, 87% *e.e.*) was obtained after purification of **19b** through a silica gel chromatography column (eluent: CH_2_Cl_2_). ^1^H-NMR (400 MHz, CDCl_3_): δ = 1.88–2.04 (m, 8H), 2.17 (t, *J* = 12.9 Hz, 1H), 2.47 (d, *J* = 14.3 Hz, 1H), 4.05 (s, 3H), 6.04 (s, 1H), 6.86–6.88 (m, 3H), 7.12–7.14 (m, 3H), 7.30–7.33 (t, *J* = 7.6 Hz, 1H), 7.45–7.50 (m, 2H), 7.57-7.61 (m, 2H), 7.67 (d, *J* = 8.1 Hz, 1H), 7.87–7.92 (m, 2H), 8.03 (s, 1H), 8.65 (d, *J* = 8.7 Hz, 1H), 8.75 (s, 1H). ^13^C-NMR (100 MHz, CDCl_3_): δ = 33.4, 44.7, 48.9, 55.9, 56.3, 82.4, 104.7, 116.8, 124.4, 125.1, 125.4, 125.5, 127.0, 127.4, 127.9, 128.0, 128.9, 129.8, 129.9, 130.1, 130.5, 131.6, 133.4, 134.7, 141.1, 142.2, 156.7 ppm. HRMS (ESI): [M + H]^+^, found 555.1769. C_33_H_33_BrNO_2_^+^ requires 555.1728. [Astec^®^ Cyclobond^®^ I 2000 RSP (250 mm × 4.6 mm, 5 µ), MeOH:(50 μM CH_3_COONH_4_ aqueous solution) = 80%:20%, flow = 0.8 mL, T = 20 °C, λ = 225 nm, t_R_ = 6.3 min (**TM-05-a**), 8.3 min (**TM-05-c**)].

*(1S,2S)-1-(7-Bromo-3-methoxynaphthalen-2-yl)-4-(dimethylamino)-2-(naphthalen-1-yl)-1-phenylbutan-2-ol* (**TM-05-d**). **TM-05-d** (0.1 g, 23.8%, Rf = 0.25, CH_2_Cl_2_/MeOH = 50:1, 97% *e.e.*) was obtained after purification of **19b** through a silica gel chromatography column (eluent: CH_2_Cl_2_). ^1^H-NMR (400 MHz, CDCl_3_): δ = 1.93–2.06 (m, 8H), 2.32 (t, *J* = 12.0 Hz 1H), 2.49 (d, *J* = 14.3 Hz, 1H), 3.00 (s, 3H), 5.86 (s, 1H), 6.46 (s, 1H), 7.20–7.25 (m, 4H), 7.34–7.42 (m, 3H), 7.51–7.55 (m, 2H), 7.77 (d, *J* = 8.1 Hz, 1H), 7.86 (s, 1H), 7.91-7.97 (m, 3H), 8.34 (s, 1H), 8.52 (d, *J* = 8.7 Hz, 1H). ^13^C-NMR (100 MHz, CDCl_3_): δ = 35.0, 42.0, 43.7, 54.4, 54.9, 104.6, 116.6, 124.0, 124.9, 125.7, 126.5, 127.4, 127.5, 128.6, 128.7, 128.9, 129.1, 129.2, 129.4, 129.6, 129.7, 131.1, 131.2, 131.4, 134.3, 138.1, 139.1, 155.2 ppm.

## 4. Conclusions

Asymmetric synthesis and AC assignment were achieved for our bedaquiline analogues with a TARB skeleton. Comparison of theoretical and experimental ECD spectra is a very useful and reliable tool to determine the AC and is especially suitable for our analogues, which are not very flexible and have many chromophoric groups. The vicinal diol intermediate revealed its critical role for our AC confirmation due to the special reactivity of the vicinal diol moiety. Moreover, our results consolidated the generality of Sharpless epoxidation. Our NOESY spectra and existing crystallographic data on the (1*R*, 2*R*) isomer of **TM-05** strongly indicate that the replacement of naphthalene did not alter the conformation of bedaquiline too much, which is a reasonable explanation for our early bioactivity results. Further research to find the most bioactive isomers of our analogues is ongoing.

## Supplementary Materials

Supplementary materials can be accessed at: http://www.mdpi.com/1420-3049/20/12/19846/s1.
