# Supplementary material for: Asymmetric Synthesis and Absolute Configuration Assignment of a New Type of Bedaquiline Analogue
Source: Molecules. 2015 Dec 11;20(12):22272–85. doi: 10.3390/molecules201219846 (PMC6331863; doi:10.3390/molecules201219846)
Supplement: Supplementary file 1 [file molecules-20-19846-s001.pdf]

Asymmetric Synthesis and Absolute Configuration Assignment of a New Type of Bedaquiline Analogue

Chang-Jiang Qiao, Xiao-Kui Wang, Fei Xie, Wu Zhong and Song Li

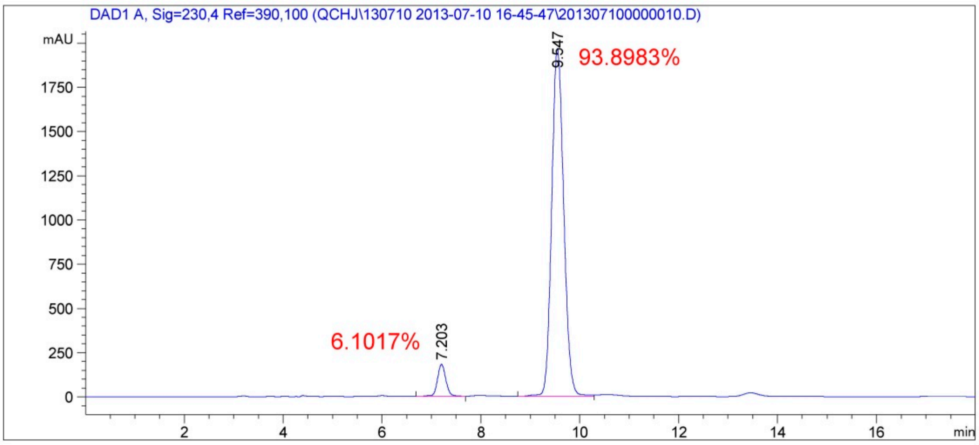

Figure S1. HPLC spectrum of **9a**, Chiralpak IC (250 mm × 4.6 mm, 5 μ), IPA/hexane = 30%:70%, flow = 1.0 mL, T = 20 °C, λ = 225 nm.

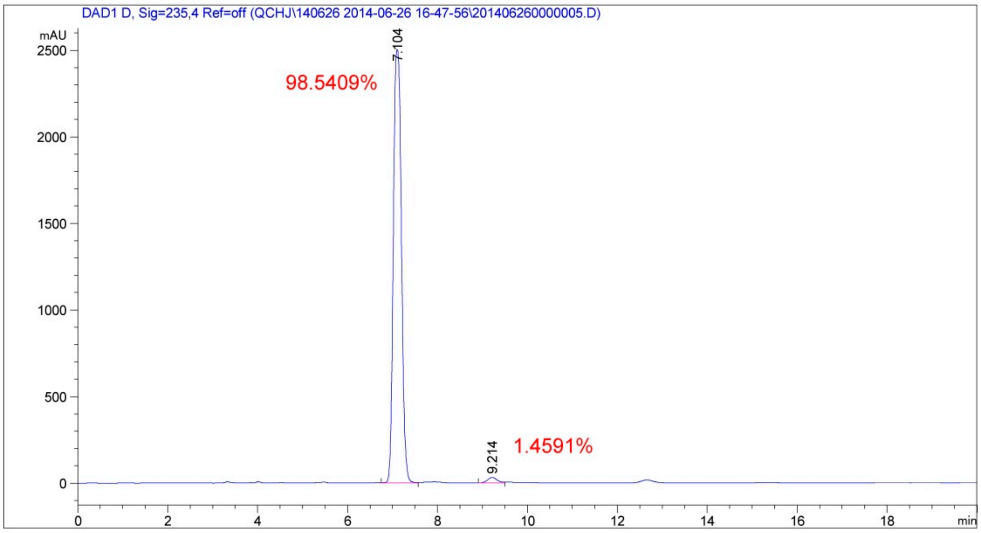

Figure S2. HPLC spectrum of **9b**, Chiralpak IC (250 mm × 4.6 mm, 5 μ), IPA/hexane = 30%:70%, flow = 1.0 mL, T = 20 °C, λ = 225 nm.

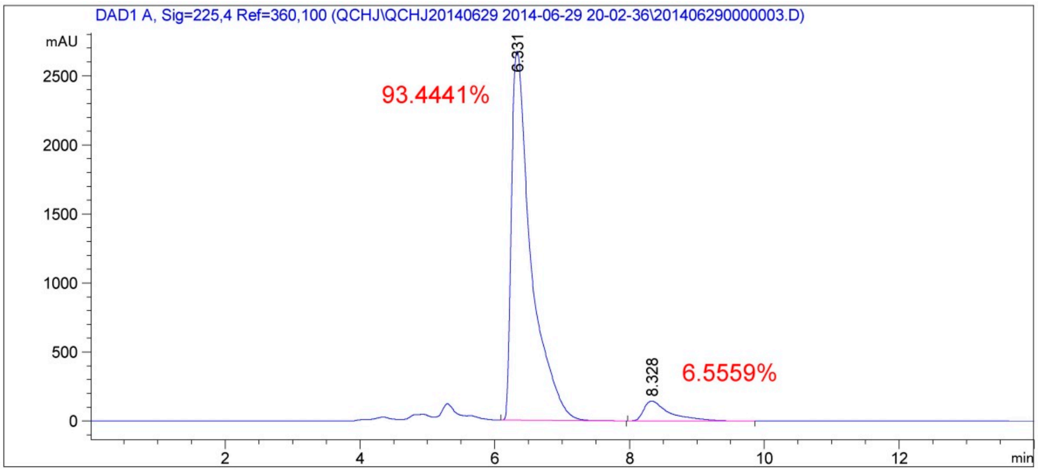

Figure S3. HPLC spectrum of **TM-05-a**, Astec® CYCLOBOND® I 2000 RSP (250 mm × 4.6 mm, 5 μ), MeOH:(50 μM CH_3COONH_4 aqueous solution) = 80%:20%, flow = 0.8 mL, T = 20 °C, λ = 225 nm.

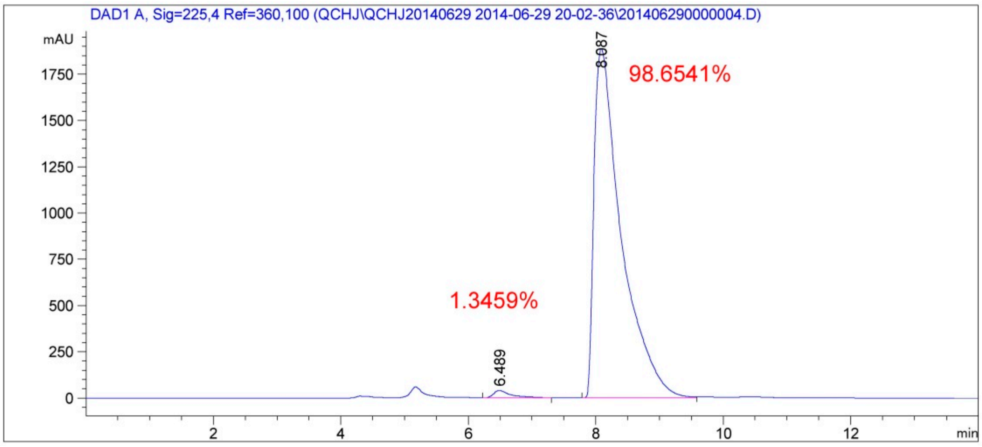

Figure S4. HPLC spectrum of **TM-05-c**, Astec® CYCLOBOND® I 2000 RSP (250 × 4.6 mm, 5 μ), MeOH:(50 μM CH_3COONH_4 aqueous solution) = 80%:20%, flow = 0.8 mL, T = 20 °C, λ = 225 nm.

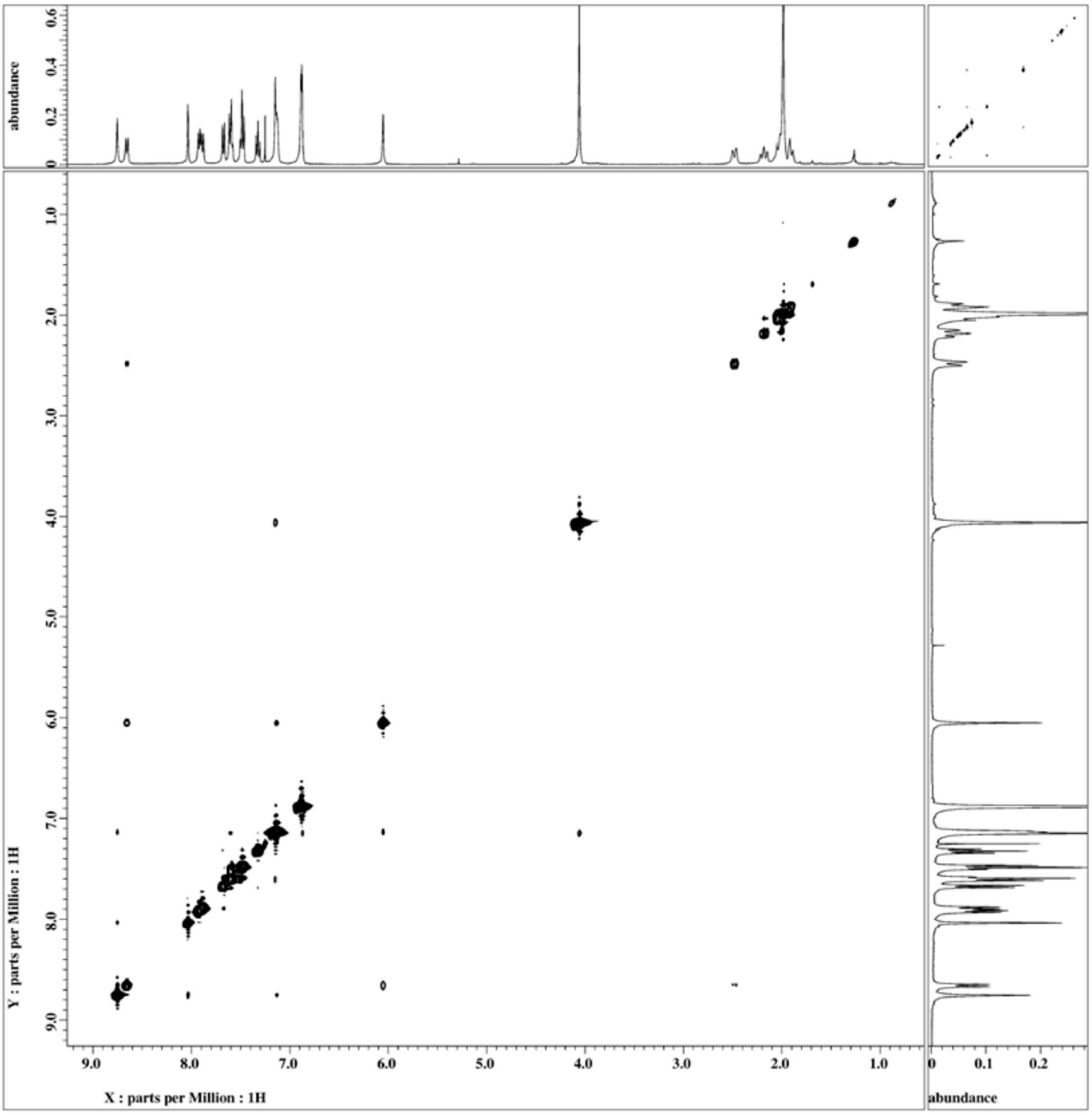

Figure S5. 2D NOESY spectrum of **TM-05-c** in $CDCl_3$.

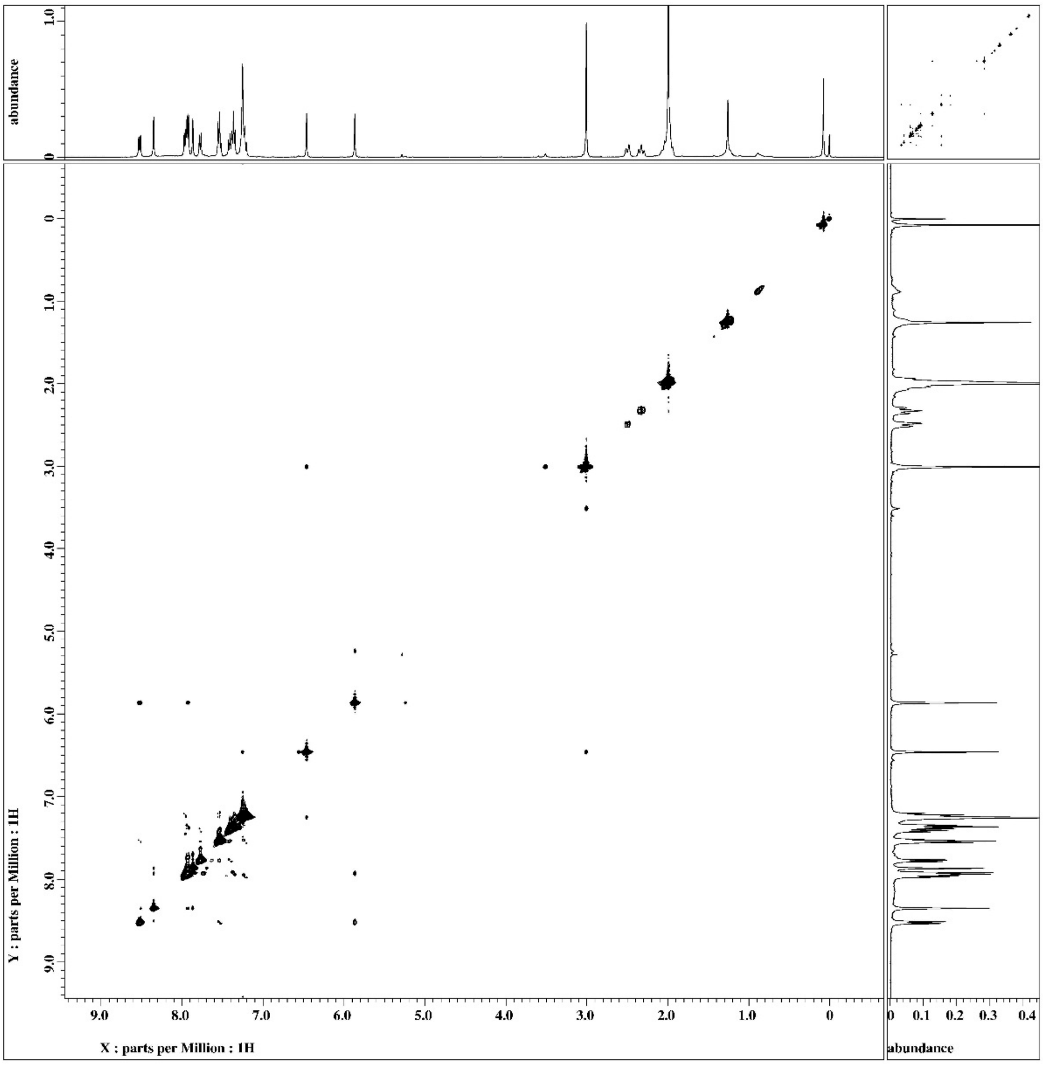

Figure S6. 2D ROESY spectrum of **TM-05-d** in $CDCl_3$

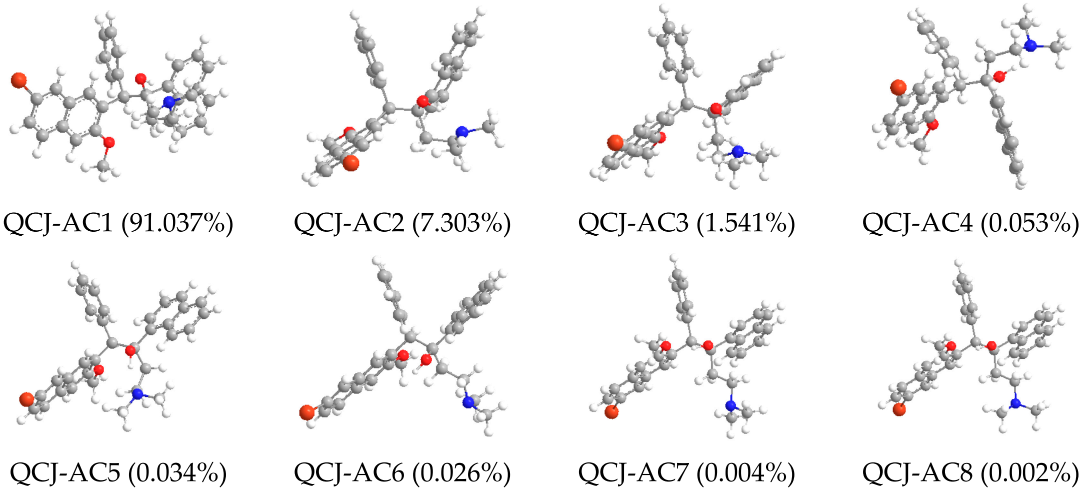

Figure S7. Computer searched eight conformers of (1*R*, 2*S*) isomer of **TM-05**.

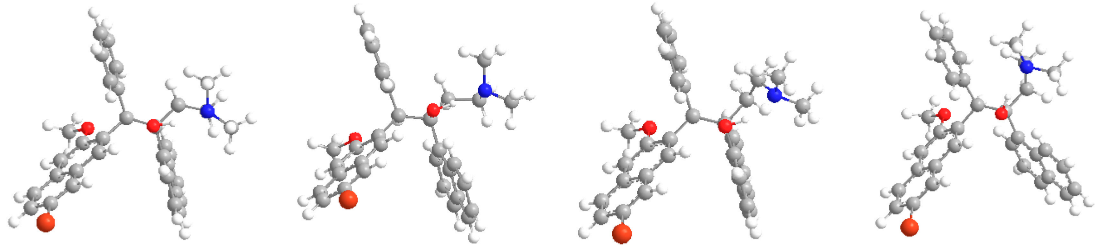

QCJ-CC1 (85.203%) QCJ-CC2 (13.722%) QCJ-CC3 (0.992%) QCJ-CC4 (0.071%)

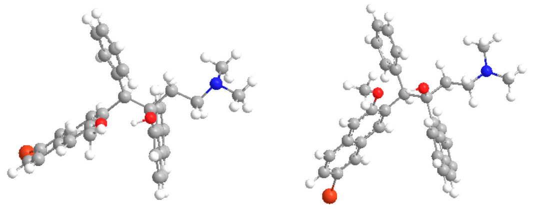

QCJ-CC5 (0.007%) QCJ-CC6 (0.005%)

Figure S8. Computer searched six conformers of (1*R*, 2*R*) isomer of **TM-05**.

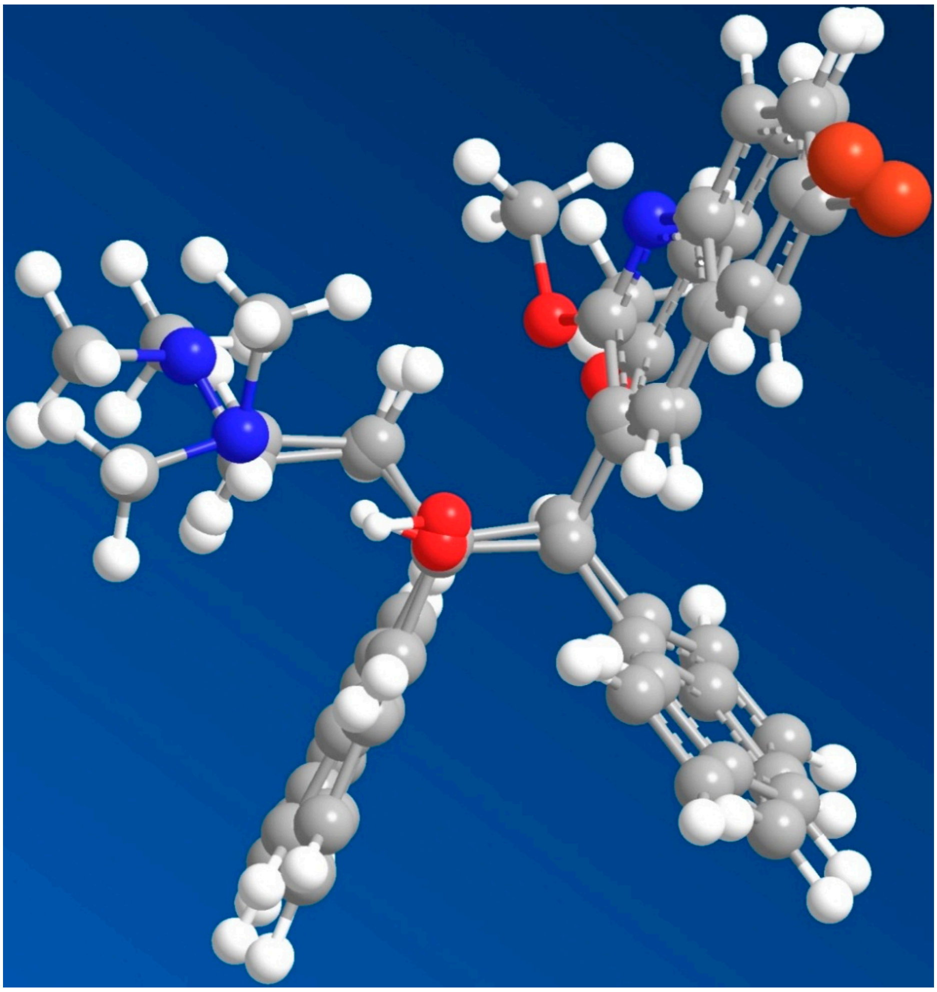

Figure S9. The overlay results between crystal structure of bedaquiline from PDB database (ID: 4V1F) and optimized conformer of (1*R*, 2*S*) isomer of **TM-05**. Fast overlay was performed on Chem3D Ultra 10.0 and the optimized conformer of (1*R*, 2*S*) isomer used was QCJ-AC1 from the calculated result in Figure S7.

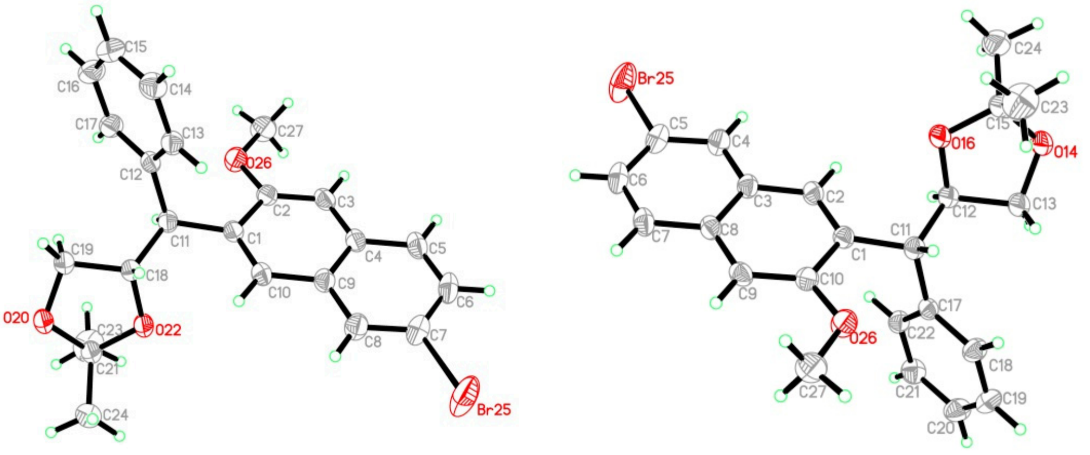

(**a**) (**b**)

Figure S10. Crystal structures of the two stereoisomers of compound **10**. (**a**) An ORTEP plot of (2*R*, 3*R*) stereoisomer of compound **10**; (**b**) An ORTEP plot of (2*S*, 3*S*) stereoisomer of compound **10**.
